# Obstructive sleep apnea syndrome promotes the progression of aortic dissection* via* a ROS- HIF-1α-MMPs associated pathway

**DOI:** 10.7150/ijbs.34888

**Published:** 2019-10-23

**Authors:** Wanjun Liu, Wenjun Zhang, Tao Wang, Jinhua Wu, Xiaodan Zhong, Kun Gao, Yujian Liu, Xingwei He, Yiwu Zhou, Hongjie Wang, Hesong Zeng

**Affiliations:** 1 Division of Cardiology, Department of Internal Medicine, Tongji Hospital, Tongji Medical College, Huazhong University of Science and Technology, Wuhan, 430030, PR China; 2 Hubei Key Laboratory of Genetics and Molecular Mechanisms of Cardiological Disorders, Wuhan, 430030, PR China; 3 Department of Cardiology, Affiliated Hospital of Weifang Medical University, Weifang, Shandong, 261000, PR China; 4 Department of Forensic Medicine, Tongji Medical College, Huazhong University of Science and Technology, Wuhan, 430030, PR China

**Keywords:** Aortic dissection, OSAS, HIF-1α, KC7F2, ROS, MMP2/9

## Abstract

**Aims:** Obstructive sleep apnea syndrome (OSAS) has been increasingly recognized as an independent risk factor for aortic dissection (AD) and it is strongly associated with the extent of intermittent hypoxia and re-oxygenation (IH). This study aimed to clarify role of ROS- HIF-1α-MMPs pathway in the pathogenesis of AD and whether the HIF-1α inhibitor attenuates AD formation.

**Methods and results:** 8-week-old male ApoE-/- mice were given β-aminopropionitrile at a concentration of 0.1 % for 3 weeks and infused* via* osmotic mini pumps with either saline or 2,500 ng/min/kg angiotensin II (Ang II) for 2 weeks. To mimic the OSAS, one group was exposed to IH, which consisted of alternating cycles of 20.9% O2/8% O2 FiO2 (30 episodes per hour) with 20 s at the nadir FiO2 during the 12-h light phase, 2 weeks before Ang II infusion. After Ang II infusion, we assessed remodeling in the aorta by echocardiography, histological and immunohistochemical analysis. IH treatment resulted in significant enlargement of the luminal area, destruction of the media, marked thickening of the adventitia, higher incidence of AD formation and lower survival rate in compared with the Ang II only group. Moreover, IH exposure markedly increased the aortic ROS production and subsequent HIF-1α expression, which in turn promoted the expressions of VEGF, MMP2 and MMP9 and finally leading to the progression of AD. Besides,* in vitro* study confirmed that IH induced HIF-1α expression plays an important role in the induction of MMPs and that is regulated by the PI3K/AKT/FRAP pathway. Intriguingly, a selective HIF-1α inhibitor KC7F2 could significantly ameliorate IH exposure induced aforementioned deleterious effects* in vitro and in vivo.*

**Conclusion:** OSAS induced IH can promote the occurrence and progression of AD* via* a ROS- HIF-1α-MMPs associated pathway. The selective HIF-1α inhibitor KC7F2 could be a novel therapeutic agent for AD patient with OSAS.

## 1. Introduction

Obstructive sleep apnea syndrome (OSAS) is a common but underestimated potentially dangerous disease, the prevalence is estimated to range from 3% to 7% in the general population but may be much higher^1^-^4^. Pathophysiologically, the disease is characterized by repetitive complete or partial upper airway collapse, resulting in interruption of airflow and persistence of inspiratory effort during sleep. Despite increasing breathing efforts, the upper airway collapse results in episodes of obstructive hypopneas or apneas affecting the sleep architecture and the whole body* via* instant and long-term mechanisms^1^,^4^.

Several meta-analyses have recently concluded that OSAS, particularly moderate-to-severe OSAS, increases all-cause mortality as well as cardiovascular events^5^-^8^. In 2003, Sampol* et al.* first showed that patients with thoracic aortic dissection (AD) presented a high prevalence of previously undiagnosed and frequently severe OSAS^9^. Since then, observational studies consistently reported that OSAS is highly prevalent among patients with aortic aneurysm and AD^10^. Several mechanisms are discussed regarding the link between OSAS and aortic disease: 1) nocturnal negative intrathoracic pressure surges leading to mechanical stretching of the aorta and ultimately aortic distension; 2) arousal-induced reflex sympathetic activation with subsequent hypertension; 3) intermittent hypoxia and re-oxygenation (IH) associated with autonomic nervous system activation and 4) consequently increased oxidative stress^8^-^12^.

Moreover, Clinical and experimental data have confirmed that IH induced reactive oxygen species (ROS) and hypoxia inducible transcription factor-1 (HIF-1) contribute majorly to the deleterious consequences in the cardiovascular diseases^13^-^15^. Therefore, it is quite reasonable to minimize the deleterious effects in AD patients with OSAS by ameliorating IH.

Continuous positive airway pressure (CPAP) is a well-known standard treatment for OSAS, effective CPAP treatment presents yet a considerable clinical challenge, as reported in the literature only about 30-60% of patients are adherent to CPAP^16^. Besides, whether CPAP therapy can reduce the incidence of AD in patients with OSAS remain controversial 8, so that new therapies, such as pharmacological approach may be another alternative. It has been reported that HIF-1 associated transcriptional network contributes to the pathogenesis of atherosclerosis, abdominal aortic aneurysm formation, pulmonary hypertension and systemic hypertension associated with OSAS^17^, ^18^. Thus, HIF-1 pathway, especially interference of the major effector HIF-1α may be a therapeutic target to reduce IH induced deleterious effects in AD patients with OSAS. But many of the HIF-1α inhibitors have unwanted off-target side effects, or have poor pharmacologic properties, which urges scientists to seek for better inhibitors for HIF-1α^19^. Through the screening of a natural product like chemical compound library, Narita T.* et al.* identified a novel small molecule KC7F2, which markedly inhibited HIF-1α mediated transcription in cells derived from different tumor types, including glioma, breast, and prostate cancers^20^. The higher selectivity and potency of KC7F2 makes it an optimal candidate for our current study. Despite currently available clinical findings, there is so far no animal study to elucidate the role of OSAS in AD. To this end, within the current study, we established an angiotensin II induced AD plus IH mouse model to mimic the AD patient with OSAS. The mechanism(s) by which OSAS affects AD has been further investigated with the help of the newly established OSAS-AD* in vivo* mouse model and* in vitro* cell culture studies.

Finally, as the HIF-1 pathway is amendable to treating interventions, we evaluated the therapeutic potential of the selective HIF-1α inhibitor KC7F2 in the OSAS-AD mouse model.

## 2. Materials and Methods

See supplementary methods for further details.

## 3. Result

### 3.1 IH treatment exacerbated Ang II-induced AD Formation in ApoE-/- Mice

Since the AD mouse model varies widely in the published literature, a pilot study was executed, in which different concentrations of AngII minipump with or without BAPN treatment were tested in 8 weeks old male ApoE^-/-^ mice, finally, the 14 days of 2,500 ng/min/kg Ang II with 21 days of BAPN treatment group has the highest incidence of AD among the tested groups (Figure S1A-B), thus this model was chosen for the following experiment.

**Figure 1:**
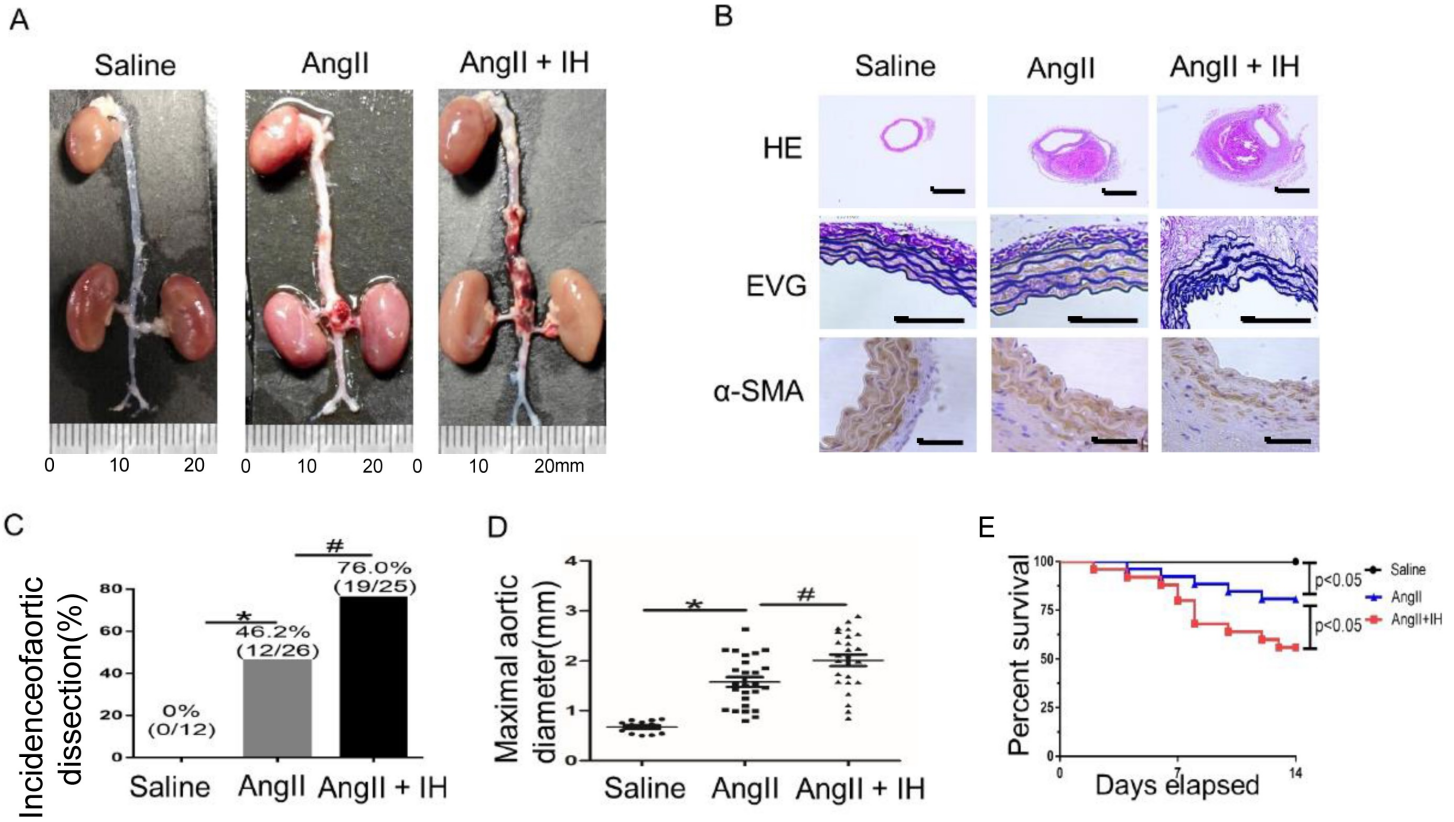
** IH treatment exacerbated Ang II-induced AD Formation in ApoE^-/-^ Mice. (A)** Representative images of aortas isolated from ApoE^-/-^mice. Aortas were treated with vehicle for 14 days (saline), Ang II alone for 14 days (Ang II), or IH treatment for 28 days and then Ang II for 14 days (Ang II+IH). **(B)** HE, α-SMA immunohistochemical and elastin van Gieson staining of aortas with different interventions are shown. IH treatment significantly exacerbated Ang II-induced aortic elastin degradation and α-SMA disorganization. **(C-D)** IH significantly enhanced the incidence of AD and exacerbated maximal abdominal aortic diameter enlargement induced by Ang II infusion. Mean± SEM, n=12 mice in saline group, n=26 mice in Ang II group and n= 25 mice in Ang II+IH group. (*p<0.05 vs.saline; #p <0.05 vs. Ang II, t-test). **(E)** Survival curve of treatment groups. ApoE^-/-^ mice were treated with vehicle for 14 days (saline), Ang II alone for 14 days (Ang II), or IH treatment for 14 days and then additional Ang II for 14 days (Ang II+IH). The survival rate was analyzed by Kaplan-Meier survival analysis and compared by the log-rank test; Scale bar 100μm.

First, to determine the effect of IH-induced AD formation, we exposed mice to IH for 4 weeks. Then after 14 days of Ang II or saline infusion, mice were euthanized and aortas were isolated for examining the occurrence of AD (Figure S1B). To assess the histological features of the AD, we performed HE, α-SMA immunohistochemical and elastin staining. Histologic examination revealed marked enlargement of the luminal area, destruction of the media and marked thickening of the adventitia in the Ang II group after 14 days. Moreover, these changes were exacerbated by IH treatment (Figure 1A-B, Figure S2A). Ang II-infusion resulted in an incidence of AD (46.2%, 12/26) in ApoE-/-mice. Ang II-infusion also resulted in a higher maximal aortic diameter (1.58±0.10 mm) in ApoE-/- mice than in control mice (0.67±0.034 mm). IH treatment markedly enhanced the incidence of AD (76.0%, 19/25) and the maximal aortic diameter (2.01±0.11 mm) induced by Ang II (Figure 1C-D). Ang II and IH treatment resulted in a higher mortality of AD (44.0%, 11/25) in ApoE-/- mice than in Ang II-infused mice (19.2%, 5/26) (Figure 1E). These results indicated that IH significantly enhanced the development of AD.

### 3.2 IH treatment enhanced aortic oxidative stress and HIF-1α expression

**Figure 2:**
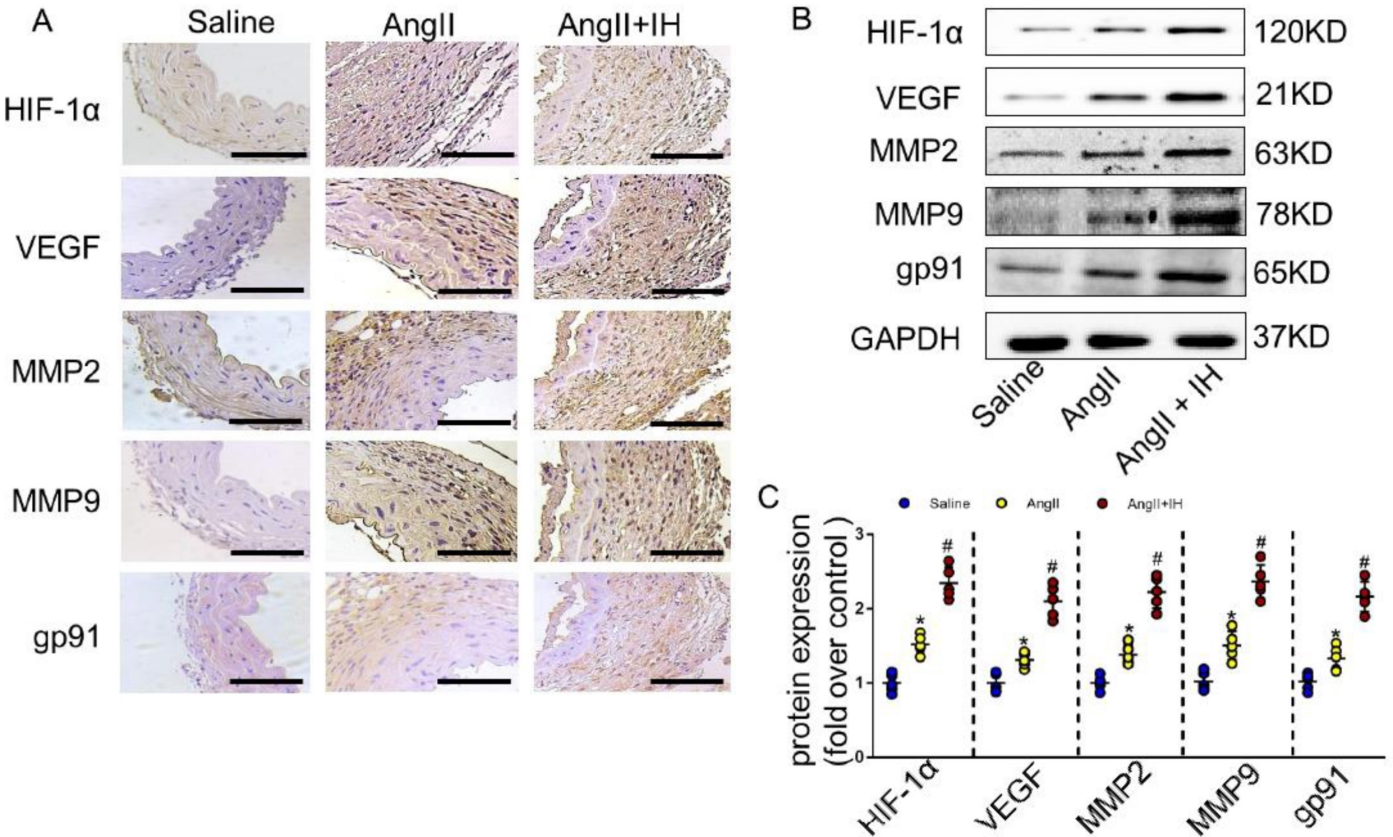
** IH treatment enhanced aortic oxidative stress and HIF-1α expression. (A)** Representative HIF-1α, VEGF, MMP2, MMP9 and the subunits of NAD (P) H gp-91 immunohistochemical staining images of aortas with indicated interventions. **(B-C)** Expression of HIF-1α, VEGF, MMP2, MMP9 and the subunits of NAD (P) H gp-91 in aortas with different interventions are shown. IH significantly exacerbated Ang II-induced aortic oxidative stress and MMPs expression as evaluated by western blotting. Mean± SEM, n=5 mice per each group (*p<0.05 vs. saline; #p<0.05 vs. Ang II, one-way ANOVA).

Growing evidence shows that reactive oxygen species (ROS) play a crucial role in the occurrence and development of AD^21^. We therefore examined the activation status of ROS-associated NAD (P) H subunits gp91. Additionally, we also used dihydroethidium staining to detect the location of superoxide production within aortic tissue. Immunohistochemical staining (Figure 2A, Figure S2B) and western blotting (Figure 2B-C) showed that IH exposure markedly aggravated aortic activity of ROS and expressions of gp91, which were substantially induced by Ang II infusion. Besides, IH exposure could further promote the ROS production in cultured VSMCs* in vitro*, as illustrated by the Dihydroethidium staining (Figure S3). HIF-1α is a major transcriptional factor in the cellular response to hypoxia, which associated with many vascular diseases^13^-^15^. Thus, we evaluated the expression levels of HIF-1α mRNA, HIF-1α protein as well as HIF-1 activity in aortic tissue by Ang II infusion and IH treatment. And as expected, exposed to IH markedly enhanced the HIF-1α expression induced by Ang II (Figure 2A-C, Figure S2B). Moreover, western blot and histologic analysis showed Ang II led to a substantial expression of VEGF, MMP2 and MMP9, which remarkably increased by IH exposure (Figure 2A-C, Figure S2B). In line with the animal study, the expression of HIF-1α was significant higher in human AD samples compared with the control group as evidenced by western blot analysis and immunohistochemical staining (Figure S4A-D), which implies that HIF-1α could be an important player in the occurrence and development of AD and a potential therapeutic target for AD treatment.

### 3.3 HIF-1α inhibitor treatment decreased MMP-2 and MMP-9 expression *in vitro*

**Figure 3:**
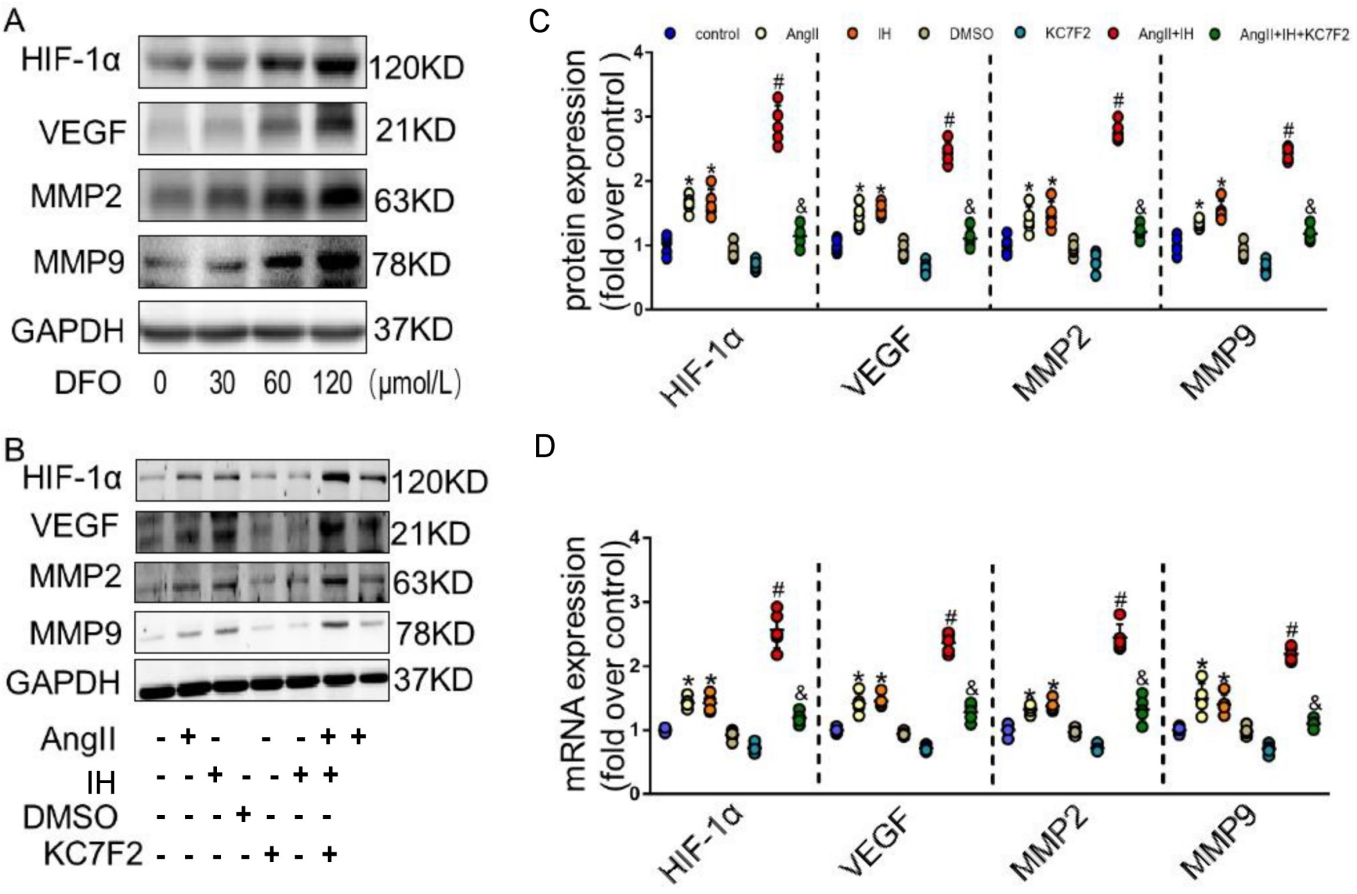
** HIF-1α inhibitor treatment decreased MMP-2 and MMP-9 expression *in vitro*. (A)** DFO induced overexpression of HIF-1α dose-dependently upregulated the expression of VEGF, MMP-2 and MMP-9 in VSMCs. The gels had been run under the same experimental conditions. **(B)** Western blotting shows the expression of VEGF, MMP-2 and MMP-9 were positive correlated with the HIF-1α expression, which was induced by Ang II, IH and their combination. While pretreating VSMCs with the select HIF-1α inhibitor KC7F2 (40μM) for 1 h could significantly suppress the HIF-1α protein expression and subsequent VEGF, MMP-2 and MMP-9 protein expression. **(C)** Scatter plot summarized the results in (B). **(D)** Real Time PCR showed the same mRNA expression tendency between groups. Mean±SEM, (*p<0.05 vs. the control group; #p<0.05 vs. the Ang II group; &p<0.05 vs. the Ang II+IH group). Multiple comparisons in (C-D) made by one-way ANOVA.

Given the observation that HIF-1α expression is induced in AD, we hypothesized that HIF-1α participates in the development of AD. We next examined effectors that are involved in the metabolism of the extracellular matrix (ECM). Evidence supports HIF-1α is a potential initiating factor in the pathway leading to MMPs' up-regulation, which play a critical role in the pathogenesis of AD. DFO, as a prolyl hydroxylase inhibitor, stabilized HIF-1α and augmented MMPs activities. As expected, DFO could cause HIF-1α accumulation by inhibiting PHD in cultured VSMCs (Figure 3A). Moreover, overexpression of HIF-1α dose-dependently up-regulated the expression of VEGF, MMP-2 and MMP-9 (Figure 3A). To further study the role of HIF-1α in AD* in vitro*, we firstly did the dose and time response experiments for HIF-1α expression induced by Ang II in cultured VSMCs (Figure S5A-B). In consistent with the previous* in vivo* findings, we found that Ang II promotes the protein expression of HIF-1α in cultured VSMCs, IH treatment further strengthen its effect. We then tested whether the pharmacological HIF-1α inhibitor could attenuate Ang II- and IH-induced upregulation of VEGF, MMP-2 and MMP-9. We found that Ang II treatment caused significant increases in VEGF, MMP-9 and MMP-2 expressions in VSMCs, and IH exposure markedly aggravated MMPs activities (Figure 3B-C). However, this effect was substantially suppressed by the addition of HIF-1α inhibitor KC7F2 (Figure 3B-C and Figure S6). We also tested the level of mRNA expression, and the results were consistent with the protein expression (Figure 3D).

### 3.4 HIF-1α inhibitor ameliorates Ang II and IH-induced MMPs' expression via the PI3K/AKT associated pathway

**Figure 4:**
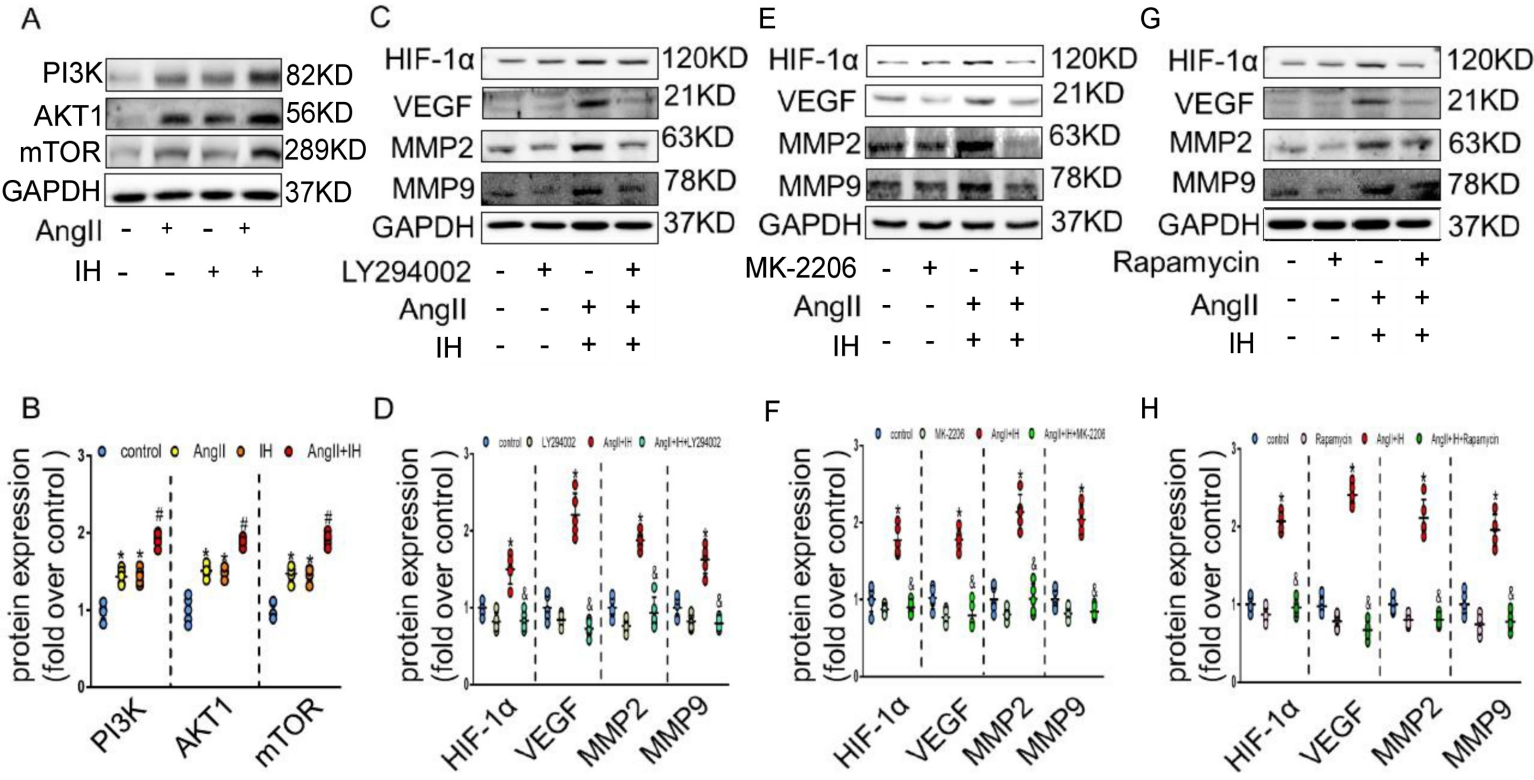
** HIF-1α inhibitor ameliorates Ang II and IH-induced MMPs expression via the PI3K/AKTFRAP pathway.** (A) Western blotting shows the expression of PI3K, AKT and mTOR were positive enhanced, which was induced by Ang II, IH and their combination. (B) Scatter plot summarized the results in (A). (B) Western blotting shows the expression of HIF-1α and MMPs. With indicated interventions, VSMCs were treated with PI3K inhibitor LY294002 for 1 hour, then incubated with Ang II and IH for 24 hours. LY294002 reduced Ang II and IH-induced HIF-1α, VEGF and MMPs expression. (D) Scatter plot summarized the results in (C). (E) AKT inhibitor MK-2206 treatment significantly blunted the deleterious effects on HIF-1α and MMPs expression induced by Ang II and IH. (F) Scatter plot summarized the results in (E). (G) VSMCs were treated with FRAP inhibitor rapamycin for 1 hour, then incubated with Ang II and IH for 24 hours. Rapamycin reduced Ang II and IH-induced HIF-1α and MMPs expression. (H) Scatter plot summarized the results in (G). Mean±SEM, (*p<0.05 vs. the control group; #p <0.05 vs. the Ang or IH group &p <0.05 vs. the Ang II+IH group, one-way ANOVA).

We next tried to identify the cellular mechanism responsible for the expression of HIF-1α by IH. As expected, AngII could promote the activation of PI3K, AKT and mTOR, which could be further enhanced under IH condition in cultured VSMCs (Figure 4A-B). To determine whether PI3K pathway activity was required for HIF-1α expression, VSMCs were exposed to LY294002, an inhibitor of PI3K, to MK-2206, an inhibitor of AKT or to rapamycin, an inhibitor of FRAP, a signaling molecule downstream of PI3K^22^. Ang II -induced HIF-1α expression was completely inhibited in the presence of LY294002, MK-2206 or rapamycin (Figure 4C-H). Taken together, these results suggest that Ang II and IH induced HIF-1α expression in VSMCs is highly dependent on PI3K activity. AKT lies between PI3K and FRAP in this signaling pathway.

### 3.5 Pharmacological HIF-1α inhibition ameliorates the progression of established AD *in vivo*

**Figure 5:**
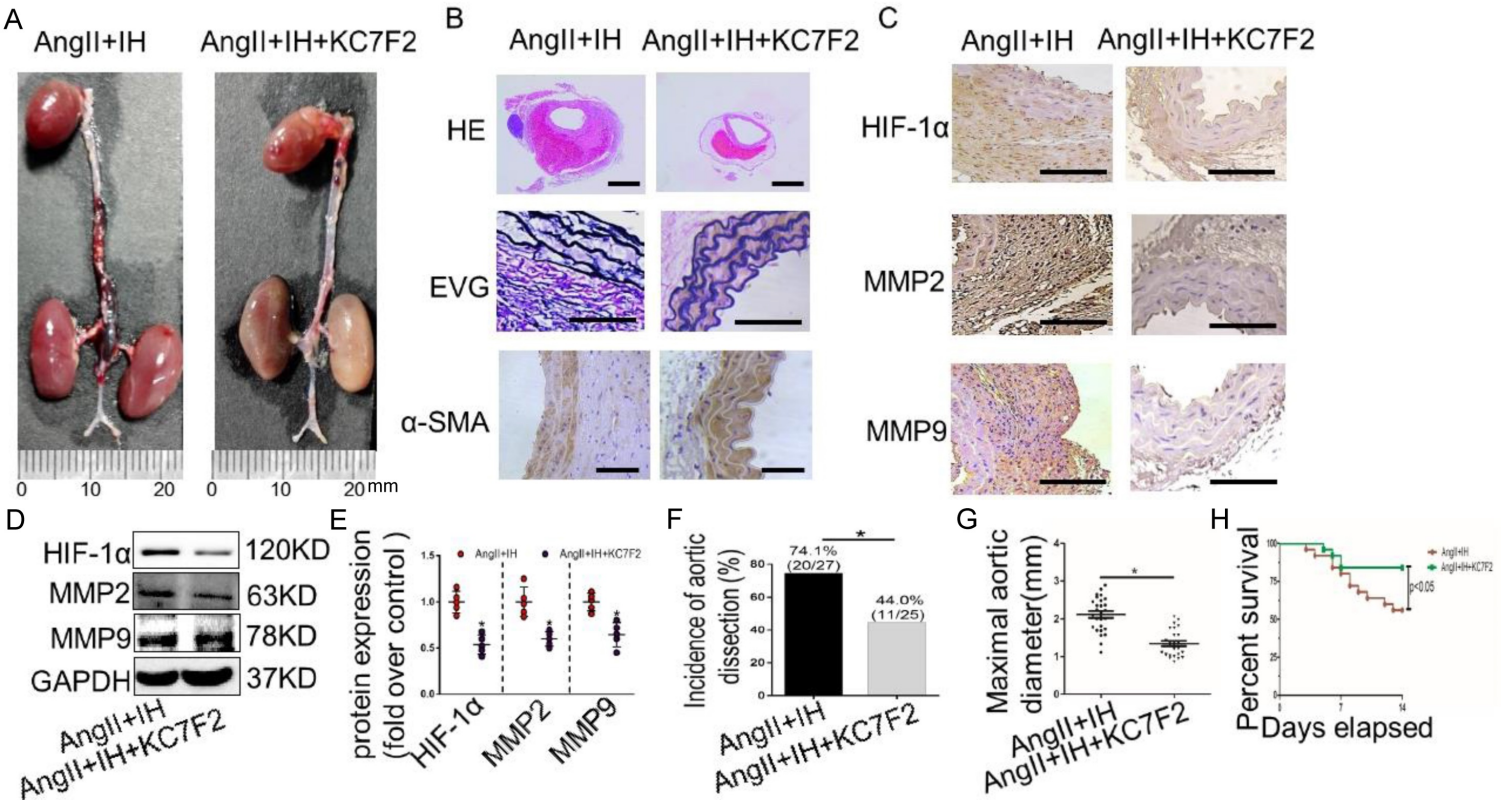
** Pharmacological HIF-1α inhibitor treatment limits the progression of established AD (A)**Representative images of aortas isolated from mice with indicated interventions. IH treatment for 14 days and then additional Ang II for 14days (Ang II+IH) or plus KC7F2 treatment every other day during the IH treatment (Ang II+IH +KC7F2). **(B)** HE, α -SMA immunohistochemical and elastin van Gieson staining of aortas with different interventions were shown. KC7F2 significantly attenuated Ang II and IH-induced aortic elastin degradation and α-SMA disorganization. **(C)** Representative HIF-1α, MMP2 and MMP9 immunohistochemical staining images of abdominal aortas with indicated interventions. **(D)** Western blotting shows that KC7F2 treatment significantly blocked the Ang II and IH induced HIF-1α expression and subsequent MMPs' expression. **(E)** Scatter plot summarizes the results in (D) (*p<0.05, Ang II+IH group vs. the Ang II+IH+KC7F2 group, t-test). **(F-G)** KC7F2 significantly reduced the incidence of AD and attenuated maximal abdominal aortic diameter enlargement induced by Ang II infusion and IH treatment (*p<0.05, Ang II+IH group vs. the Ang II+IH+KC7F2 group, t-test). (H)Survival curve of the above treatment groups. Mean±SEM, n= 27 mice in Ang II +IH group and n= 25 mice in Ang II+IH+KC7F2 group (*p<0.05, Ang II+IH group vs. the Ang II+IH+KC7F2 group). The survival rate was analyzed by Kaplan-Meier survival analysis and compared by the log-rank test. Scale bare 100μm.

To examine the translational potential of HIF-1α inhibition in existing AD, treatment with the inhibitor KC7F2 or saline alone was initiated 14 days before Ang II infusion and continued until sacrifice (Figure S1C). Surprisingly, we found that KC7F2 treatment significantly improved the outcomes of the established AD in our OSAS-AD mouse model in compared with the saline treated group. In details, histologically KC7F2 treatment resulted in significant reduction of the luminal area, preservation of the media and thinning of the adventitia (Figure 5A). Besides, KC7F2 treatment preserved severe disruption of medial architecture with prominent elastin degradation in the AD tissues (Figure 5B, Figure S2C). Moreover, administration of KC7F2 significantly decreased Ang II and IH mediated HIF-1α, MMP-2 and MMP-9 expression in the aortas (Figure 5C-D, Figure S2D). In line with the above findings, KC7F2 treatment resulted in significantly less AD incidence (74.1% vs. 44.0% in KC7F2 treatment group, *p < 0.05*), much smaller maximal aortic diameter (2.11±0.09 vs.1.35± 0.07 in KC7F2 treatment group, *p< 0.05*) and sharply reduced mortality (44% vs. 16% in KC7F2 treatment group, *p< 0.05*) (Figure 5G-H).

**Figure 6:**
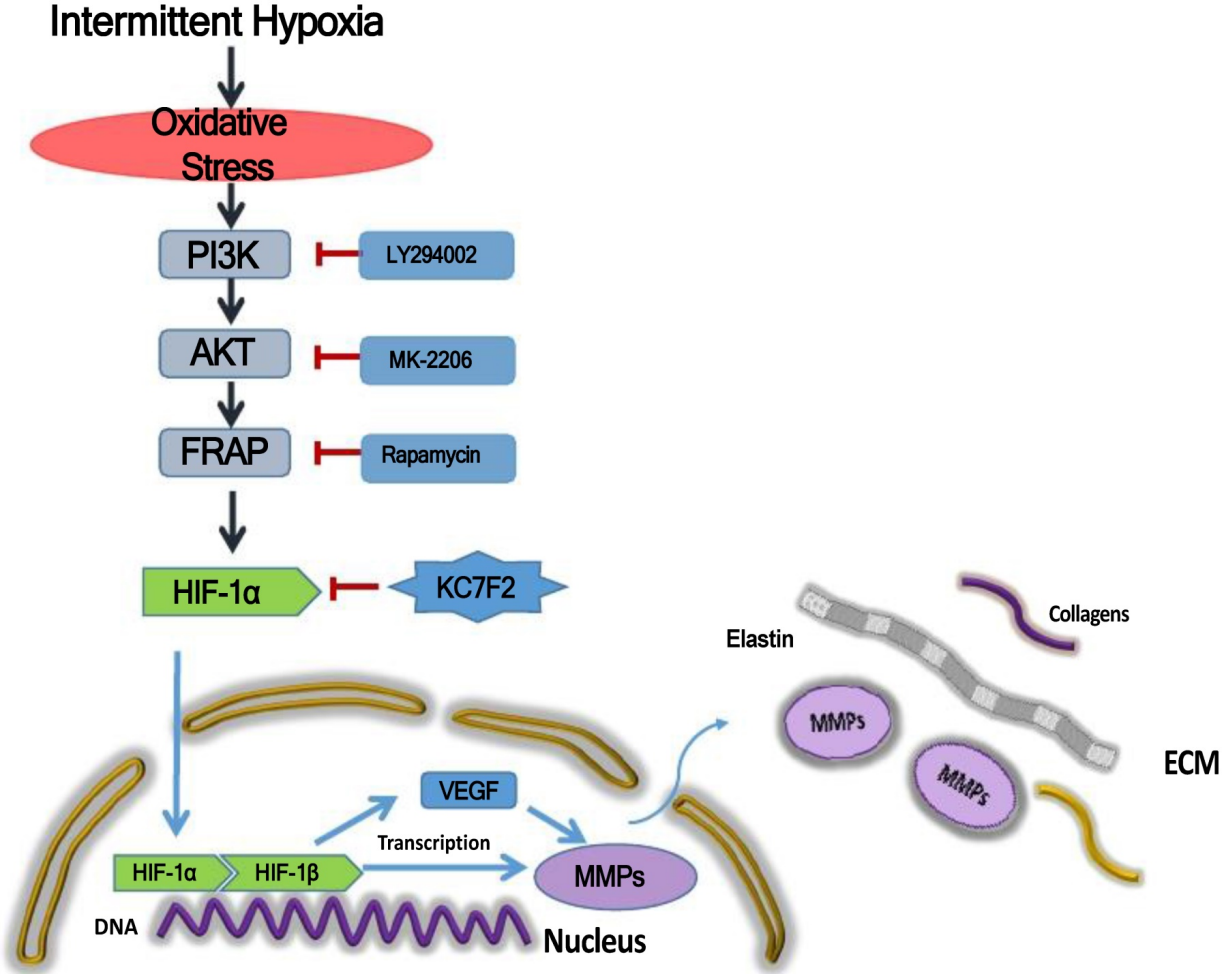
** Schematic illustration summarizes the main study findings IH exposure results in robust ROS production and ROS in turn induces HIF-1α expression via a PI3K-AKT-FRAP associated pathway.** Subsequently, stabilized HIF-1α translocates into the nucleus, together with the other transcription factors, such as HIF-1β, evoking numerous gene transcription, including VEGF and MMP-2/9, which play important role in ECM remodeling. The secreted MMPs result in aortic ECM components, like elastins and collagens, degradation, thus contributing to the occurrence and progression of AD. Pharmacological inhibition of HIF-1α by a novel small molecular inhibitor KC7F2 could ameliorate IH exposure induced deleterious effects in our OSAS-AD mouse model.

Taken together, the above results indicate that IH exposure could promote the occurrence and progression of AD and HIF-1α plays a pivotal role in between. In detail, IH exposure results in robust ROS production and ROS in turn induces HIF-1α expression* via* a PI3K-AKT-FRAP associated pathway. Subsequently, stabilized HIF-1α translocate into the nucleus, together with the other transcription factors, such as HIF-1 β , evoking numerous gene transcription, including VEGF and MMP-2/9, which play important role in ECM remodeling. The secreted MMPs result in aortic ECM components, like elastins and collagens, degradation, thus contributing to the occurrence and progression of AD. Pharmacological inhibition of HIF-1α by a novel small molecular inhibitor KC7F2 could ameliorate IH exposure induced deleterious effects in our OSAS-AD mouse model, which potentiates its clinical application for AD patients with OSAS in the future (Figure 6).

## 4. Discussion

A potential pathophysiological role of the OSAS in AD has been reported by several clinical studies^8^-^12^. However, the exact mechanisms remain unsolved, especially the animal study is still lacking, majorly owing to the unavailability of a proper experimental mouse model. Within the current study we established the first time a novel OSAS-AD mouse model, which could recapitulate the phenotype of AD patient with comorbidity of OSAS.

Thanks to the newly established OSAS-AD experimental mouse model, we could show that the OSAS is a vital risk factor, contributing to the progression and rupture of AD* via* a ROS- HIF-1α-MMPs associated pathway. Moreover, blocking the aforementioned signaling pathway by a selective HIF-1α inhibitor KC7F2 could ameliorate the progression and rupture of AD under OSAS state* in vivo*, rendering KC7F2 a novel potential therapeutic agent^20^. Since OSAS is an independent risk factor for cardiovascular diseases(CVD) such as arterial hypertension, myocardial infarction, atherosclerosis, heart failure, arrhythmia and stroke, chronic intermittent hypoxia re-oxygenation (IH) has been widely applied as a useful animal model for studying OSAS related CVD^14^, ^23^-^28^. Fletcher EC* et. al.* established in the early 1990s an IH rat model, in this model male rats were held in a hypoxia chamber and subjected to intermittent hypoxia (3-5% nadir ambient oxygen) every 30 seconds, 7 hours per day for up to 35 days.

Thereafter they found that OSAS could chronically increase the blood pressure as well as the left ventricle-to-body weight ratio^25^. In another rat IH model a cycle of 40 s of hypoxia (5% oxygen) and 20 s of normoxia (21% oxygen) was executed 8 hours each day for 14 consecutive days. And the authors claimed that OSAS could increase both the blood pressure and infarct size in an acute myocardial infarction model^14^. Similarly, in a mouse IH model, two-month and 18-month old mice were subjected to IH (20% O2 40 sec-6% O2 20 sec) 6 hours per day for 6 weeks. Finally, IH increases in macroscale stiffness of left ventricle myocardium extracellular matrix, suggests that the ECM plays a role in the cardiac dysfunction induced by OSAS^29^. Moreover, in order to closely mimic the chronicity of human OSAS, Cortese R *et. al.* analyzed the saturation recordings of adult patients with moderate to severe OSAS and made a better mouse IH profile, which consists of 90 sec of 6.1% O2 balance nitrogen alternating with 90 sec 21% O2 throughout the 12 hours of light time (07:00 a.m.-07:00 p.m.) and normoxia for the remaining 12 hours of nighttime, and lasts for 20 weeks. With this new IH mouse model, they showed that OSAS, in absence of concurrent pro-atherogenic settings (i.e., genetic propensity or dietary manipulation) leads to the recruitment of CD36(+) high macrophages to the aortic wall and trigger atherogenesis^26^. As reported in the literature IH has been used in most of the CVD models related to OSAS, but the experimental mouse model mimicking AD patient with OSAS is still lacking. By reviewing and analyzing the published data, in our current study we establish an OSAS-AD mouse model, by combining the IH receipt which recapitulates the adult patients with moderate to severe OSAS and a modified Ang II induced AD mouse model which has been developed in our laboratory^26^, ^30^. In this newly developed mouse model, we exposed 8-week-old male ApoE-/- mice to IH with a paradigm consisted of alternating cycles of 20.9% O2/8% O2 FiO2 (30 episodes per hour) with 20 sec at the nadir FiO2 during the 12 hours light phase (07:00 a.m.-07:00 p.m.), and normoxia for the remaining 12 hours of nighttime, and lasts for 4 weeks, and the Ang II minipump were implanted 2 weeks after the IH was initiated (As illustrated in Figure S2A). All mice were kept on a regular low-fat chow diet. After evaluating this new mouse model, we were able to show that compared with the control group the IH group has a higher incidence of AD formation, a larger maximal aortic diameter and a lower cumulative survival rate. These phenotypical data are in consistent with the human findings^8^-^12^. Hence, we establish a reliable and easy-to-use mouse model for investigating the role of OSAS in AD. With the newly established OSAS-AD experimental mouse model, we investigated the molecular mechanism(s) underlying OSAS promoting the formation and progression of AD. It has been widely recognized that chronic IH, a major component of OSAS, contributes to OSAS related aortic disease through increasing ROS production and oxidative stress^10^.

Furthermore, a great number of transcription factors and signaling pathways are modulated by ROS, one of the most prominent and relevant to OSAS being hypoxia inducible factor-1α (HIF-1α)^14^. HIF-1α is a major transcriptional factor in the cellular response to hypoxia. Recent data show that transcriptional factor HIF-1α is a master regulator of oxygen homeostasis, playing critical roles in physiological and pathological processes, such as apoptosis, erythropoiesis, angiogenesis, senescence, adhesion, differentiation, proliferation and survival^15^. Moreover, HIF-1α is a positive regulator of multiple key pathological processes involved in abdominal aortic aneurysm formation, including oxidative stress, MMPs elaboration, SMC apoptosis and vascular inflammation^31^-^36^. Previous studies demonstrate that the combination of HIF-1α, VEGF and MMP-2 may serve a molecular predictor for lymph node metastasis, which also indicate that the HIF-1α/VEGF signaling pathway may be associated with MMP-2 expression^37^, ^38^. Based on the above findings, we hypothesized that IH could promote the formation and progression of AD* via* a ROS-HIF-1α-MMPs dependent pathway. Indeed, consistent with the literature, in the current study immunohistochemical staining and western blot detected more expression of Gp91, HIF-1α, VEGF and MMPs (2 and 9) in aortae of OSAS-AD mice compared with respective control. Interestingly the expression of the HIF-1α is more VSMC prone in the middle layer of the aortae, suggesting an important role of VSMC in this signal pathway.

Subsequent* in vitro* studies using cultured VSMCs further supported the important role of HIF-1α in regulating MMPs and HIF-1α inhibition by a novel small molecule KC7F2 could restrict the HIF-1α induced expression of VEGF and MMPs (2 and 9)^20^. However, we could not exclude the role of other participating cells in AD, such as barrier endothelial cells and other immune cells, such as T lymphocyte, B lymphocyte, dendritic cells, monocytes/macrophages and* etc.*, these requires further investigation, e.g. producing HIF-1α cell specific knock out mice. It has been demonstrated that nitric oxide and tumor necrosis factor induce HIF-1α* via* the PI3K/AKT pathway^39^, ^40^, likewise, activation of PI3K/AKT/FRAP pathway participates in HIF-1α induction in response to serum and epidermal growth factor treatment^41^. Our study further revealed that Ang II preferentially activates PI3K/AKT, a critical pathway responsible for HIF-1α protein synthesis^42^. Importantly, our results showed that PI3K, AKT and FRAP inhibitors effectively blocked the Ang II-induced increase in HIF-1α protein expression, suggesting a role for the PI3K and FRAP dependent translational machinery in Ang II mediated upregulation of HIF-1α protein levels. These results further confirm the central role of HIF-1α in promoting the formation and progression of AD under OSAS state, and HIF-1α may serve as a therapeutic target.

To explore the therapeutic potential of HIF-1α intervention for AD patients with OSAS, a novel small molecule HIF-1α inhibitor KC7F2 was tested in our OSAS-AD mouse model. Excitingly, compared with the saline treatment KC7F2 could significantly decrease the incidence of AD formation, reduce the maximal aortic diameter and increase the cumulative survival rate in the OSAS-AD mice^20^. KC7F2 has been shown to prevent the activation of HIF-target genes such as the upregulation of fibronectin and MMP-2 and the suppression of E-cadherin and tissue inhibitor of metalloproteinases-2 in human liver cancer cells^43^, which is in line with our* in vitro* findings in VSMC. Although the protective effect of KC7F2 is potent, its mechanism remains still incomplete, further gene array and/or proteomic analysis are required to fully decipher the underlying mechanisms. Besides, in the translational aspect preclinical and clinical trials are stringently needed before its clinical application.

In summary, in the current study we established a reliable and easy-to-use experimental mouse model, mimicking the AD patient under OSAS state, which could be a valuable tool for investigating the role of OSAS in AD in the future. Furthermore, we confirmed that the OSAS contributes to the formation and progression of AD* via* a ROS- HIF-1α-MMPs associated model. Finally, the HIF-1α inhibitor KC7F2 ameliorated the deleterious effects in AD mice exposed to IH, which potentiates its application as a novel therapeutic agent for AD patient with moderate to severe OSAS.

## Supplementary Material

Supplementary figures and tables.

## Abbreviations

AD: aortic dissection
OSAS: Obstructive sleep apnea syndrome
IH: intermittent hypoxia
ROS: reactive oxygen species
HIF-1α: hypoxia inducible factor-1α
MMP2/9: Matrix metalloproteinase 2/9
VEGF: Vascular endothelial growth factor
Ang II: Angiotensin II
PI3K: Phosphoinositide-3-kinase
FRAP: FKBP-rapamycin associated protein
BAPN: β-aminopropionitrile
