# Supplementary material for: Obstructive sleep apnea syndrome promotes the progression of aortic dissection via a ROS- HIF-1α-MMPs associated pathway
Source: Int J Biol Sci. 2019 Oct 23;15(13):2774–82. doi: 10.7150/ijbs.34888 (PMC6909961; doi:10.7150/ijbs.34888)
Supplement: Supplementary file 1 — Supplementary figures and tables. [file ijbsv15p2774s1.pdf]

Supplementary methods

OSAS-AD mouse model

The animal procedures conform to the guidelines from Directive 2010/63/EU of the European Parliament on the protection of animals used for scientific purposes and approved by the Institutional Animal Research Committee of Tongji Medical College. $ApoE^{-/-}$ mice purchased from Beijing Vital River Laboratory Animal Technology Co., Ltd. $ApoE^{-/-}$ mice (C57BL/6 background) were housed at the animal care facility of Tongji Medical College under specific pathogen-free conditions and fed a normal diet. 8-week-old male $ApoE^{-/-}$ mice were given β-aminopropionitrile (BAPN) at a concentration of 0.1 % for 3 weeks[1, 2] and infused *via* osmotic mini pumps (Alzet, Cupertino, CA) with either saline or 2,500 ng/min/kg angiotensin II (Ang II) (Sigma-Aldrich, St. Louis, MO) for 14 days. To evaluate the effect of HIF-1α inhibitor KC7F2[3] treatment on AD initiation and progression, treatment was initiated 14 days before and terminated 14 days after Ang II infusion. KC7F2 was freshly prepared in PBS and administered to mice at a dose of 10 mg/kg every other day through intraperitoneal injection. The IH paradigm consisted of alternating cycles of 20.9% O2/8% O2 FiO2 (30 episodes per hour) with 20 sec at the nadir FiO2 during the 12-h light phase (07:00 a.m.–07:00 p.m.), as deoxygenation-reoxygenation episodes occur in moderate to severe OSAS patients. After 4 weeks of IH exposure, including 2 weeks before and

sustained 2 weeks during Ang II infusion, mice were transferred to room air, cardiac function was measured by echocardiography, and then humanely euthanized by anesthetic overdose (pentobarbital) for organ collection (Figure S1). Blood pressure was measured using the tail-cuff method described previously and after implantation, and prior to sacrifice.

Histology and immunohistochemistry

Sacrificed mice were perfused with ice-cold PBS and then with 4% buffered paraformaldehyde. Tissues were further fixed in 4% buffered paraformaldehyde for 2 days at 4°C, embedded in paraffin and processed for sectioning, 4 μm cross-sections were then obtained. Aortic morphology was evaluated using hematoxylin and eosin stained histological sections[4]. Images were captured using an LEICA DM4000B Microscope (LEICA, Beijing, China). At least 10 random images per mouse around the dissection position were taken, and the maximal diameter was picked for further analysis, at least 12 mice per group were included into each group. van Gieson Stain was performed according to the manufacture's description (Boster, Wuhan, China)[4]. Aortic sections were stained with Weigert Solution (Fuchsin basic, resorcinol, water, and hydrochloric acid) for 6 hours, directly immerged into Differentiation Solution (1% hydrochloric acid alcohol), and then flushed with water. Van Gieson Dye Solution (1% Fuchsinacid aqueous solution and saturated aqueous picric

acid solution) was used to restain the sections for 1-2 minutes. A microscope (LEICA DM4000B) was used to observe images. At least 5 independent samples in each group (for each sample 3 sections were obtained) were observed.

Immunohistochemical analyses of HIF-1α, VEGF, MMP2, MMP9 and GP91 was conducted essentially as previously described[5]. Paraffin embedded tissue sections were deparaffinised and rehydrated, incubated with a specific primary antibody (1 h, at room temperature), washed 3 times with PBS, and incubated with an appropriate, horseradish peroxidase-conjugated secondary antibody. Peroxidase activity was detected using a DAB substrate (3,3'-diaminobenzidine) and slides were counterstained with haematoxylin. Control images were obtained following incubation with a non-specific primary antibody and were used for background correction. All histological analyses were done by two independent blinded investigators. Images were obtained using an LEICA DM4000B Microscope (LEICA, Beijing, China) at 20x or 40x magnification.

Cell culture and *in vitro* IH model

Vascular smooth muscle cells (VSMCs) (ATCC, Manassas, VA) were cultured in 10% fetal bovine serum (Gibco, Grand Island, NY) containing Dulbecco's modification of Eagle's medium (Gibco, Grand Island, NY)

under 37 ℃ and 5% CO_2 conditions[6]. In the IH group, cells were maintained at 37 ℃ at 5% CO2 in a chamber (Oxycycler model A42, Biospherix) in which O2 levels were alternated between 21% for 5 min and 1% for 10 min, for a total of 64 cycles (18 h). After confluence, VSMCs were incubated with 10 μM Ang II (Sigma-Aldrich, St. Louis, MO) for 24 h. For intervention study, 40 μM KC7F2 (Sigma-Aldrich, St. Louis, MO) were added 1 h before Ang II treatment. Cells were exposed to deferoxamine (DFO) (0, 30, 60, 120 μM) for 24h. Cells were cultured in media containing 0.1% FBS for 20 h, followed by the addition of PI3K inhibitor LY294002 (1 μM), AKT inhibitor MK-2206 (5 μM) or FRAP inhibitor rapamycin (1 nM) 1 h before Ang II and IH treatment. Cells were harvested at various time-points or interventions after IH for RNA and protein isolation.

Western Blot Analysis

Proteins were isolated using RIPA buffer and the concentration was determined using the BCA protein assay (Thermo Fisher Scientific, Waltham, MA, USA). Aorta and cell extracts were separated by SDS/PAGE and transferred to PVDF membranes. Membranes were blocked in Tris-buffered saline with 0.1% Tween 20 with 5% non-fat dry milk or bovine serum albumin. Membranes were incubated with appropriate primary antibodies overnight at 4℃. After washing 5 times

with 1 x TBS-T membranes were incubated with an appropriate secondary peroxidase-conjugated antibody, and immunoreactive proteins were visualized using an enhanced chemiluminescence system (Tanon,Shanghai, China). The following antibodies were applied: HIF-1α, VEGF, MMP2, MMP9, and GP91 were from Epitomic (Burlingame, CA, USA); MMP2 and GAPDH (Santa Cruz Biotechnologies, CA, USA). GAPDH was used for calibration of total protein or cytosolic protein determination. Bands were quantified by densitometry using Quantity One software (Bio-Rad, Hercules, CA).

RT-PCR

Total RNA was extracted from vascular smooth muscle cells (VSMCs) with Trizol reagent (TaKaRa, Japan). Then, 750 ng RNA was added to a 20 μL reaction volume for cDNA reverse transcription using the Prime Script™ RT reagent Kit with gDNA Eraser (TaKaRa, Japan), and quantitative real-time polymerase chain reaction (qRT-PCR) was performed using the SYBR R Premix Ex Taq™ (TaKaRa, Japan) in a Step One Plus real-time PCR system (Applied Biosystems, USA). The HIF-1α, VEGF, MMP2, MMP9 and GAPDH genes were analyzed. Primer sets for selected genes were designed by TianYi Huiyuan (Wuhan, China) and their sequences are listed in Supplementary Table S1. Gene expression was quantified according to the $2^{-\triangle\triangle Ct}$ method. Message RNA levels in vascular

smooth muscle cells were expressed as fold changes relative to their respective controls.

Statistical Analysis

All data analysis was performed with the use of SPSS 13.0 statistical software. Data are reported as mean ± SEM. Depending on the nature of the data, Kaplan–Meier survival analysis, one-sample *t*-test, or two-way analysis of variance followed by the Newman-Keuls post hoc correction was used to determine significance between groups. Log-rank test, ANOVA or the Student's *t* test was used to determine statistical significance with $p<0.05$. Each experiment was done at least in triplicate.

Supplementary Table S1: Sequences of human primers used within the current study.

Gene	Forward	Reverse
HIF-1α	5′-GAACGTCGAAAAGAAAAGTCTCG-3′	5′-CCTTATCAAGATGCGAACTCACA-3′
VEGF	5′-AGGGCAGAATCATCACGAAGT-3′	5′-AGGGTCTCGATTGGATGGCA-3′
MMP2	5′-GATACCCCTTTGACGGTAAGGA-3′	5′-CCTTCTCCCAAGGTCCATAGC-3′
MMP9	5′-GGGACGCAGACATCGTCATC-3′	5′-TCGTCATCGTCGAAATGGGC-3′
GAPDH	5′-GTTCAACGGCACAGTCAAGG-3′	5′-GTGGTGAAGACGCCAGTAGA-3′

Reference

1. Fashandi AZ, Hawkins RB, Salmon MD, et al. A novel reproducible model of aortic aneurysm rupture. *Surgery* 2018;**163**:397-403.
2. Eguchi S, Kawai T, Scalia R, et al. Understanding Angiotensin II Type 1 Receptor Signaling in Vascular Pathophysiology. *Hypertension* 2018;**71**:804-810.
3. Narita T, Yin S, Gelin CF, et al. Identification of a novel small molecule HIF-1alpha translation inhibitor. *Clin Cancer Res* 2009;**15**:6128-6136.
4. Wang T, He X, Liu X, et al. Weighted Gene Co-expression Network Analysis Identifies FKBP11 as a Key Regulator in Acute Aortic Dissection through a NF-kB Dependent Pathway. *Front Physiol* 2017;**8**:1010.
5. Liu W, Wang T, He X, et al. CYP2J2 Overexpression Increases EETs and Protects Against HFD-Induced Atherosclerosis in ApoE-/- Mice. *J Cardiovasc Pharmacol* 2016;**67**:491-502.
6. Huang F, Xiong X, Wang H, et al. Leptin-induced vascular smooth muscle cell proliferation via regulating cell cycle, activating ERK1/2 and NF-kappaB. *Acta Biochim Biophys Sin (Shanghai)* 2010;**42**:325-331.

Obstructive sleep apnea syndrome promotes the progression of aortic dissection *via* a ROS- HIF-1α-MMPs associated pathway

Wanjun Liu[1,2#], Wenjun Zhang[1,2#],Tao Wang[3], Jinhua Wu[1,2], Xiaodan Zhong[1,2], Kun Gao[1,2] ,Yujian Liu[1,2], Xingwei He[1,2], Yiwu Zhou[4]， Hongjie Wang[1,2*]and Hesong Zeng[1,2*]

[1]Division of Cardiology, Department of Internal Medicine, Tongji Hospital, Tongji Medical College, Huazhong University of Science and Technology, Wuhan, 430030, PR China

[2]Hubei Key Laboratory of Genetics and Molecular Mechanisms of Cardiological Disorders, Wuhan, 430030, PR China

[3]Department of Cardiology, Affiliated Hospital of Weifang Medical University, Weifang, Shandong, 261000, PR China

[4]Department of Forensic Medicine, Tongji Medical College, Huazhong University of Science and Technology, Wuhan, 430030, PR China

***Corresponding authors:**

Hongjie Wang, Email: hongjie.wang@tjh.tjmu.edu.cn , Tel. +86-27-8369-3794, Fax: +86-27-8366-3186;

Hesong Zeng, Email: zenghs@tjh.tjmu.edu.cn, Tel. +86-27-8369-2850, Fax: +86-27-8366-3186.

#W.L. and W.Z. contribute equally to this manuscript.

Supplementary Figure 1

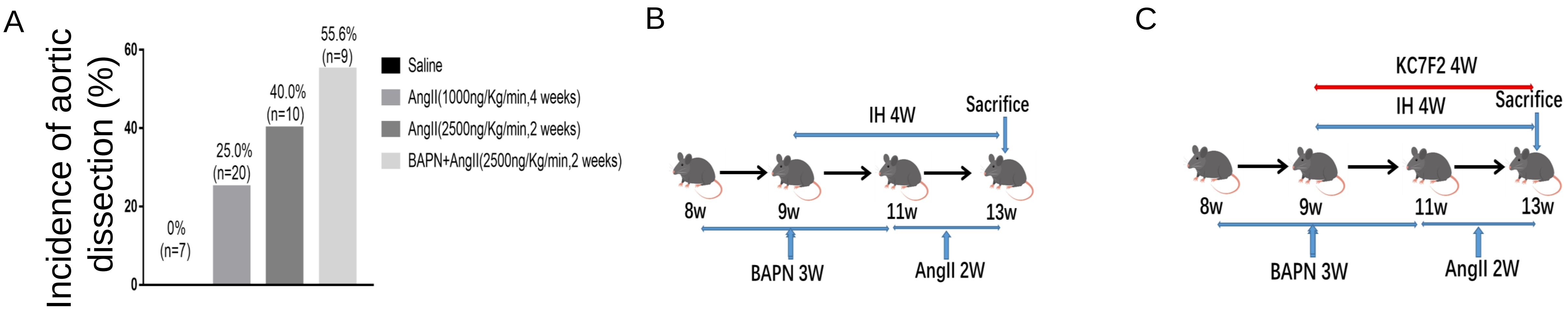

Figure S1. Schematic illustration of the OSAS-AD experimental mouse model. 8 week-old male ApoE$^{-/-}$ mice were fed on normal chow diet and infused via osmotic mini pumps with either saline or 1,000 ng/min/kg Ang II for 4 weeks , 2,500 ng/min/kg Ang II for 2 weeks; and were given BAPN at a concentration of 0.1 % for 3 weeks in the drinking water and then infused *via* osmotic mini pumps with either saline or 2,500 ng/min/kg Ang II for 2 weeks(A). 8 week-old male ApoE$^{-/-}$mice were fed on normal chow diet and were given BAPN at a concentration of 0.1 % for 3 weeks in the drinking water and then infused *via* osmotic mini pumps with either saline or 2,500 ng/min/kg Ang II for 2 weeks. The mice were exposed to IH condition from the second week of BAPN treatment and last for total 4 weeks until the Ang II treatment was finished. Finally the mice were sacrificed for further analysis at the end of Ang II treatment (B). For interventional study KC7F2 was freshly prepared in PBS and administered to mice at a dose of 10 mg/kg every other day through intraperitoneal injection during the IH period, thereafter the mice were sacrificed and analyzed(C). BAPN: β-aminopropionitrile; IH: intermittent hypoxia; Ang II: angiotensin II; KC7F2: a HIF-1α inhibitor.

Supplementary Figure 2

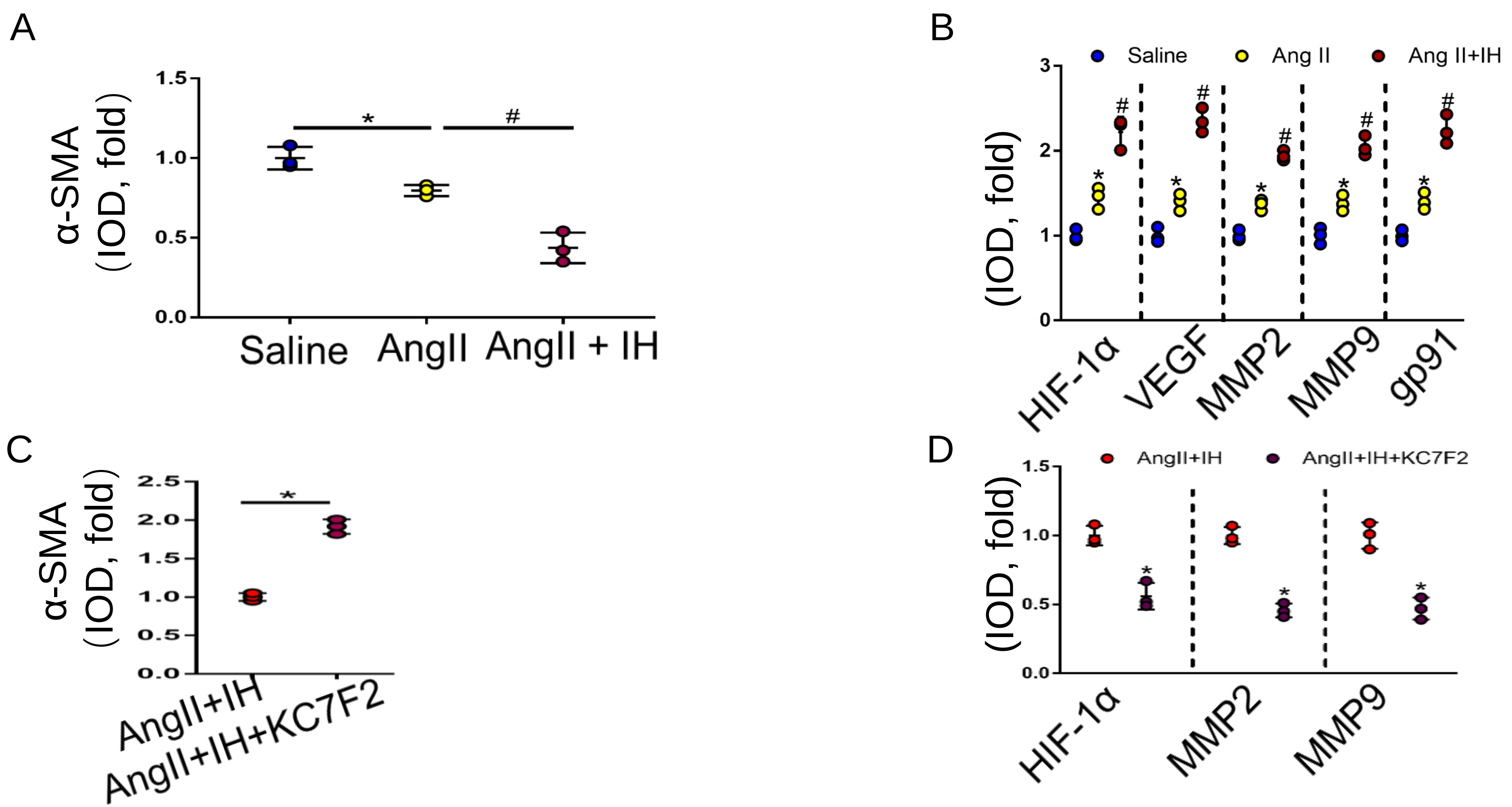

Figure S6. (A)Quantitative analysis of α-SMA.(B) quantitative analysis of HIF-1α, VEGF, MMP2, MMP9 and the subunits of NAD (P) H gp-91 expression (*p<0.05 vs. saline; #p <0.05 vs. Ang II, one-way ANOVA). (C)Quantitative analysis of α-SMA.(D) quantitative analysis of HIF-1α, MMP2, MMP9 expression (*p<0.05, Ang II+IH group *vs.* the Ang II+IH+KC7F2 group, *t*-test).Scatter plot summarized the results. All data represent the means ± SEM.

Supplementary Figure 3

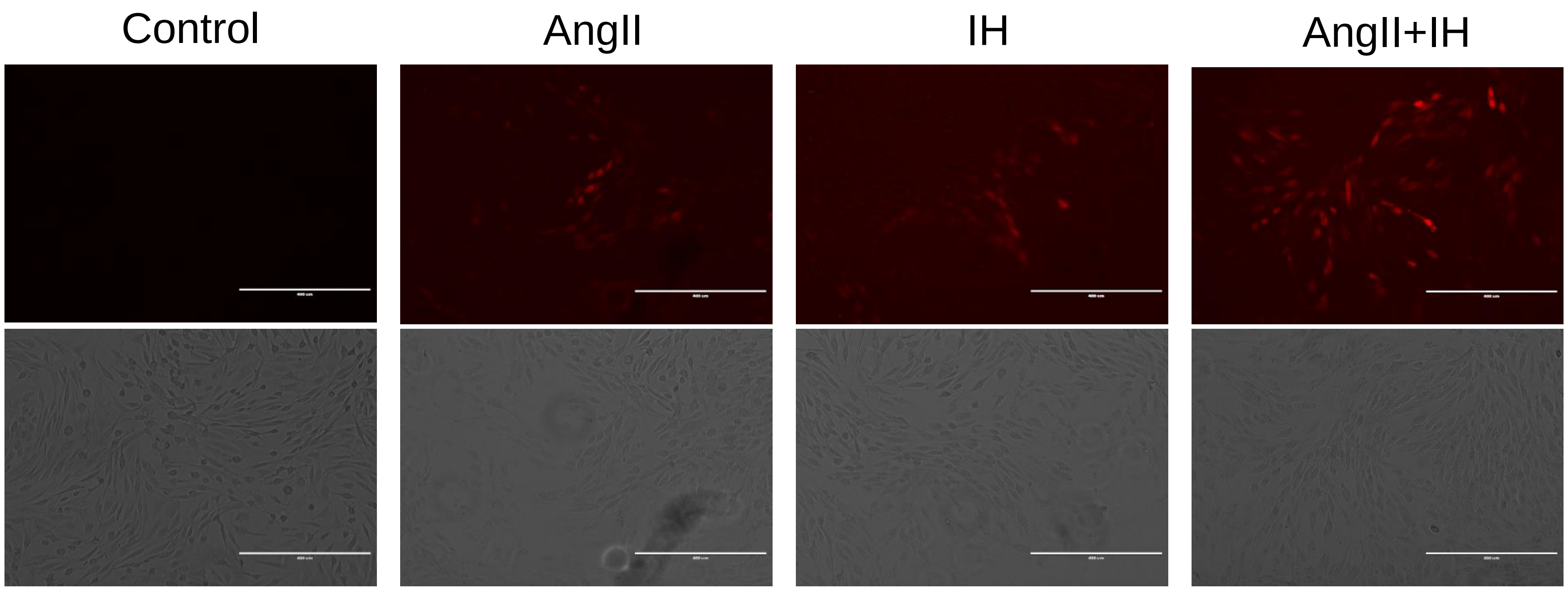

Figure S3. Ang II and IH can promote the ROS production in cultured VSMCs *in vitro*, IH on top can further increase the ROS production. Dihydroethidium staining of VSMCs were pretreated with Ang II (10 μM) or IH and both of Ang II and IH for 24 h (upper panel) , and representative light microcopy pictures for each group respectively (lower panel). Scale Bar: 400μm.

Supplementary Figure 4

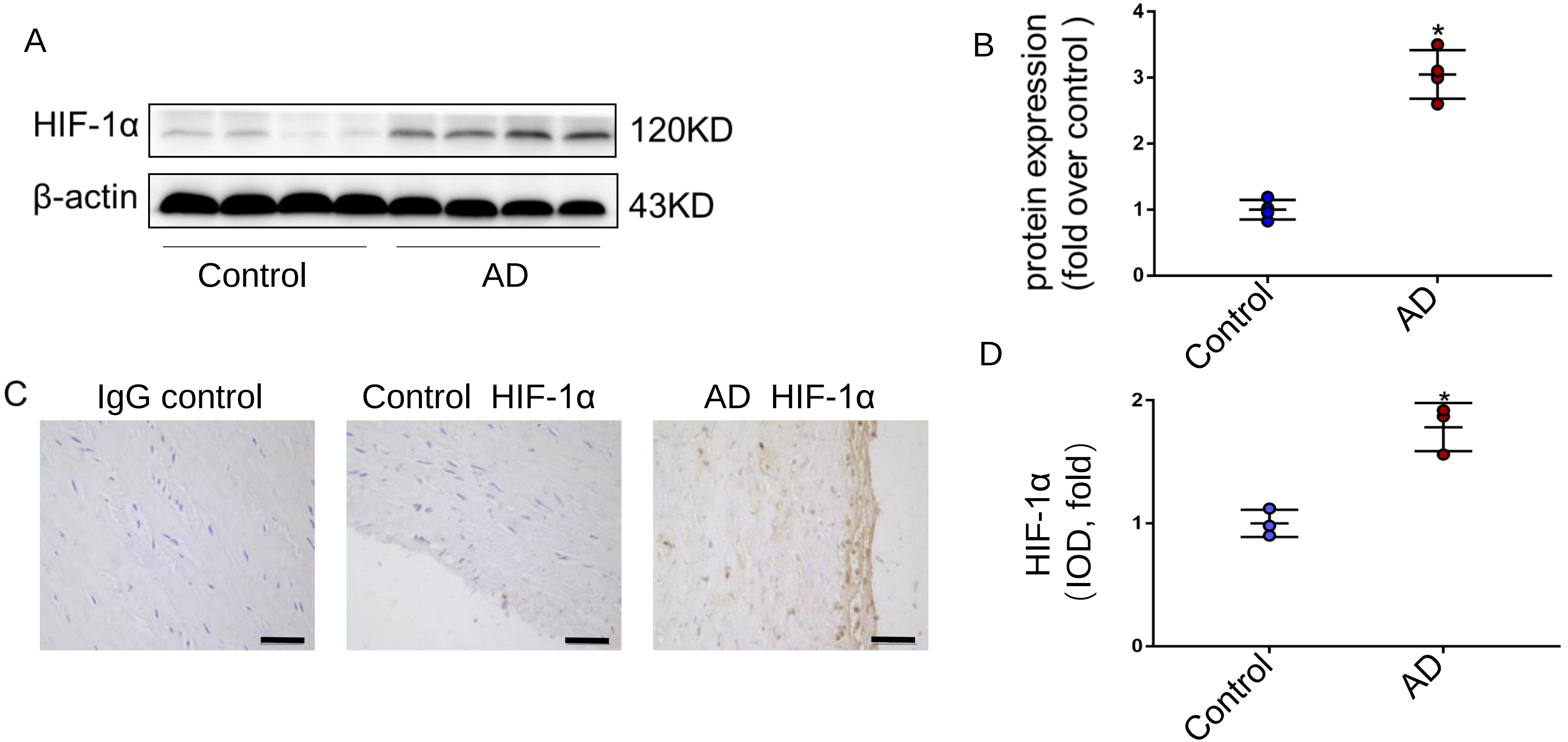

Figure S4. HIF-1α expression was upregulated in human AD samples. Western blotting shows the expression of HIF-1α in human AD tissues compared with respective control aortae(A) and Scatter plot summarized the results(B). Immunohistochemistry staining show the expression of HIF-1α in human AD tissues compared with respective control aortae (C) and Scatter plot summarized the results(C). All data represent the means ± SEM; * $p < 0.05$ *vs.* control, Scale Bar 50μm.

Supplementary Figure 5

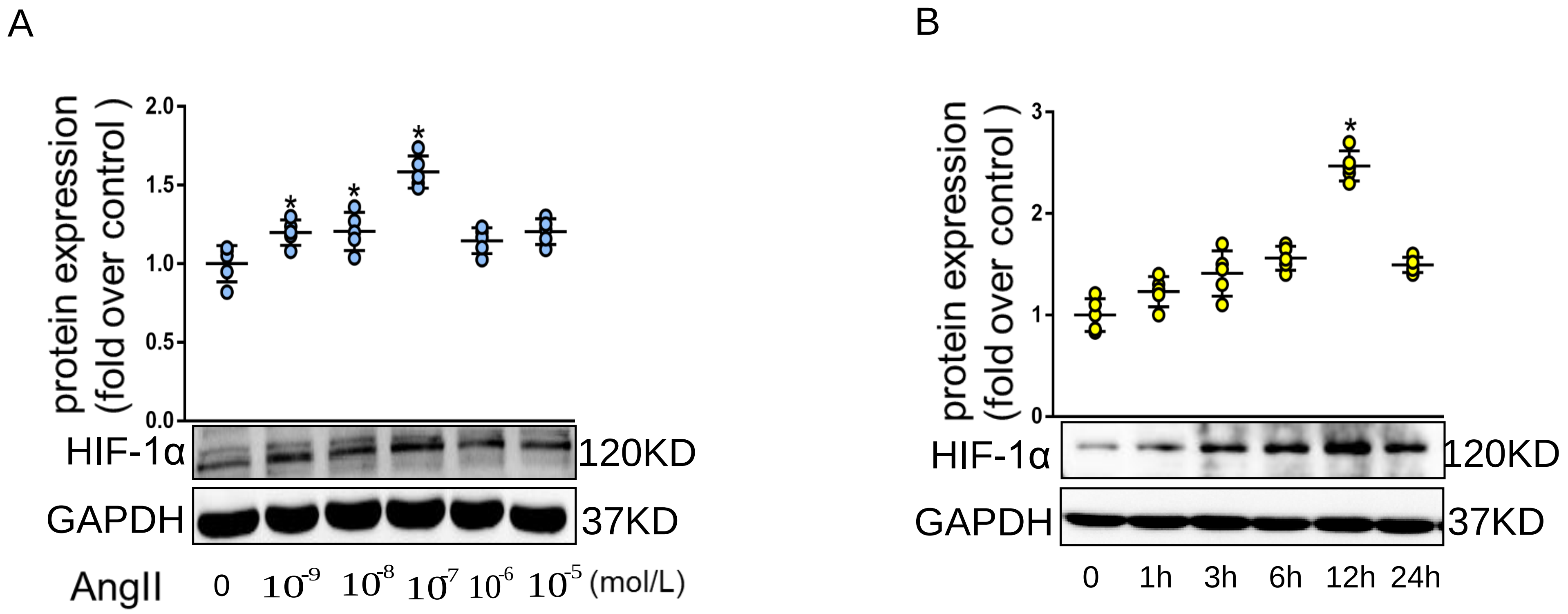

Figure S5. HIF-1α expression can be induced by Ang II treatment in cultured VSMCs *in vitro*. Western blotting shows the induction of HIF-1α by AngII in a concentration dependent (A) and time dependent (B) manner, Scatter plot summarized the results. All data represent the means ± SEM; * $p < 0.05$ *vs.* control.

Supplementary Figure 6

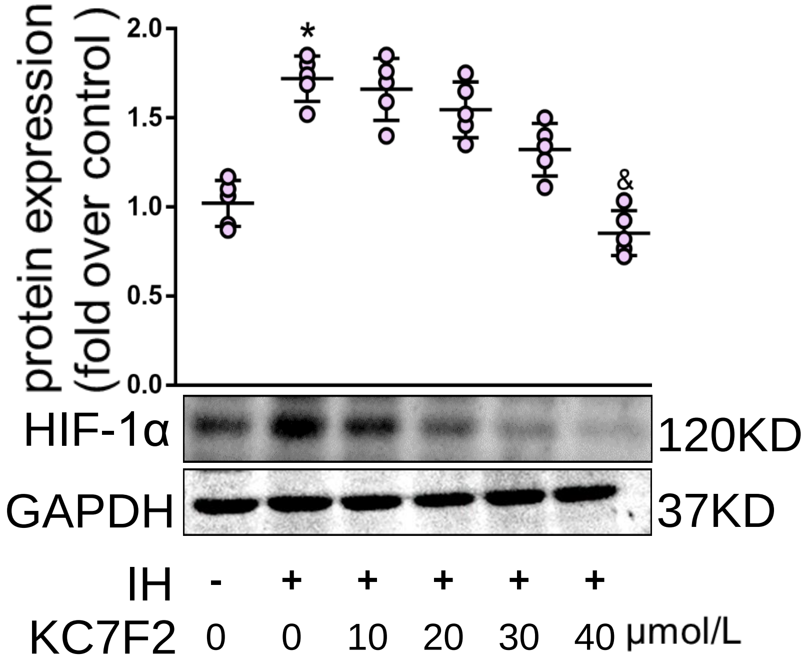

Figure S6. KC7F2 can dose dependently block the IH induced HIF-1α expression in cultured VSMCs *in vitro*. Western blotting shows the expression of HIF-1α in VSMCs can be significantly induced by IH exposure. The HIF-1α inhibitor KC7F2 can suppress the IH induced HIF-1α in a concentration dependent manner. Scatter plot summarized the results. All data represent the means ± SEM; * $p < 0.05$ vs. control, & $p < 0.05$ *vs.* IH alone.
